# Long-Term Effects of Pedicle Clamping during Major Hepatectomy for Colorectal Liver Metastases

**DOI:** 10.3390/jcm10132778

**Published:** 2021-06-24

**Authors:** Piotr Krawczyk, Marcin Morawski, Maciej Krasnodębski, Damian Sieńko, Michał Grąt, Philipp Kron, Peter Lodge

**Affiliations:** 1 Department of General, Transplant, and Liver Surgery, Medical University of Warsaw, 02-097 Warsaw, Poland; piotrek.m.krawczyk@gmail.com (P.K.); mwkrasn@gmail.com (M.K.); damiansienko7@gmail.com (D.S.); michal.grat@wum.edu.pl (M.G.); 2 Department of HPB and Transplant Surgery, St. James’ University Hospital, Leeds LS9 7TF, UK; philipp.kron@nhs.net (P.K.); peter.lodge@nhs.net (P.L.)

**Keywords:** colorectal cancer metastases, Pringle maneuver, liver resection, mortality

## Abstract

The use of the Pringle maneuver (PM) varies widely among surgical departments. Its use depends on the operator and type of liver resection. The aim of this study was to determine the impact of the PM on patient outcomes when undergoing major liver resections. This retrospective study comprised 179 colorectal liver metastasis patients from two liver centers from Leeds and Warsaw. Only right or right extended hepatectomies with negative oncological margins were included. The primary outcome measure was the 5-year overall survival (OS). The PM was applied during 60 (33.5%) major hepatectomies included in the study and was associated with a higher peak 3-day postoperative bilirubin concentration (*p* = 0.002), yet not with the peak 3-day alanine aminotransferase activity (*p* = 0.415). The 5-year OS after liver resections with the PM and without the PM were 55.0% and 33.4%, respectively (*p* = 0.019). Following stratification by the Tumor Burden Score, after resections with the use of the PM, superior survival was particularly found in the subgroup of patients at intermediate risk of recurrence (*p* = 0.004). However, the use of the PM had no significant effect on the 5-year overall survival following adjustment for the confounding effect of the carcinoembryonic antigen concentration (*p* = 0.265). The use of the PM had no negative effects on the long-term outcomes in patients undergoing major, oncologically radical liver resections for colorectal metastases.

## 1. Introduction

Liver resection is the most effective method of treatment of patients with resectable primary cancers and metastatic liver tumors. One of the major issues in liver resection is the intraoperative control of bleeding. Intraoperative blood loss is associated with increased mortality; therefore, various techniques are used to reduce parenchymal bleeding [1]. Over the years, many techniques of temporary hepatic ischemia have been proposed that rely on the selective or complete, continuous, or temporary occlusion of the hepatic vessels [2,3].

Despite the usefulness of these techniques during resection procedures and the relative safety of stopping the hepatic inflow, occlusion of blood flow to the liver and its subsequent restoration causes ischemia-reperfusion injury (IRI) [4]. The IRI is caused by a rapid inflow of blood to the previously ischemic liver, which results in impaired microcirculation and damage to hepatocytes and stimulates the synthesis of inflammatory mediators [5]. All these factors may represent one of the main causes of post-hepatectomy liver failure and potentially increase the risk of postoperative cancer recurrence [6,7]. 

In animal studies, temporary hepatic ischemia had both direct and remote negative effects on progression of liver tumors [8]. Therefore, despite its effectiveness as a method of intraoperative bleeding control during liver resection, the impact of the use of the PM on long-term outcomes of patients with colorectal liver metastases undergoing surgical treatment remains to be elucidated, particularly in the context of the potentially detrimental effects of IRI on postoperative course of the disease. 

In the present work, we present the impact of the use of Pringle’s maneuver on the long-term patient survival outcomes based on the analysis of patients from two university centers in Leeds and Warsaw.

## 2. Materials and Methods

This was a retrospective observational two-center study that included 179 patients who underwent major liver resection (right or right extended hemihepatectomy with up to two non-anatomical resections in the left liver segments) for colorectal cancer metastases in the Department of General, Transplant, and Liver Surgery, Medical University of Warsaw, Poland (119 patients), and Department of Hepatobiliary and Transplant Surgery, St James’ University Hospital, Leeds, United Kingdom (60 patients). 

The study cohort was selected from a group of a total of 505 patients who underwent major liver resection (four right liver segments or more—right or right extended hemihepatectomy with up to two non-anatomical resections in the left liver segments) between January 2009 and December 2013 at Warsaw’s (259 patients) and Leeds’ units (246 patients). Exclusion criteria comprised (1) R1 or R2 resection, (2) extrahepatic disease, (3) prior or (4) concomitant intraoperative radiofrequency ablation (RFA), and (5) portal vein embolization. The Pringle maneuver was used selectively, not on a routine basis.

Patient death was set as the primary endpoint assessed over a 5-year postoperative follow-up period. The 5-year overall survival was calculated from the day of resection to the date of death. Observations were censored at the date of last available follow-up or 5 years postoperatively, whichever occurred first. The stratifications of patients accordingly to the newly proposed Tumor Burden Score (TBS) were applied in analyses. For each patient, we calculated the TBS according to the formula: TBS^2^ = (maximum tumor diameter [cm])^2^ + (number of liver lesions)^2^ as proposed by Sasaki et al. accordingly to Zones 1, 2, and 3 using the cut-off points of <3, ≥3 to <9, and ≥9, respectively [9]. 

The patient characteristics were analyzed and compared between groups using the Mann–Whitney U test, Chi^2^ test, and Fisher’s exact test as appropriate. Cox proportional hazards regression models were used for univariable analyses to determine the risk factors for worse overall survival. Two-factor models were used to test the potential confounding effects of other covariates on the association between PM use and the overall survival. Hazard ratios (HRs) were presented with 95% confidence intervals (95% CI). The Kaplan–Meier estimator and log-rank test were applied for the survival curve analyses. The study protocol was approved by the institutional review board of the Medical University of Warsaw. The level of significance was set at 0.05. Finally, STATISTICA version 13.3, TIBCO Software Inc. (Palo Alto, CA, USA) software was used for computing the statistical analyses.

## 3. Results

The baseline characteristics of the study cohort are shown in Table 1. Regarding the extent of resection, most of the patients underwent right hemihepatectomy. The PM was utilized in 33.5% patients. Comparison of the baseline characteristics between patients undergoing liver resection with and without the PM is presented in Table 1. Notably, patients undergoing liver resection with the PM had a lower preoperative carcinoembryonic antigen concentration (CEA) (*p* = 0.026) and higher peak 3-day postoperative bilirubin concentration (*p* = 0.002) as compared to those undergoing liver resection without the PM. Other significant intergroup differences were found, including the preoperative white blood cell count, preoperative platelets count, and preoperative bilirubin concentration. Otherwise, groups were similar with respect to the baseline characteristics, particularly with respect to the number of tumors, cumulative size of tumors, and TBS. 

**Figure 1:**
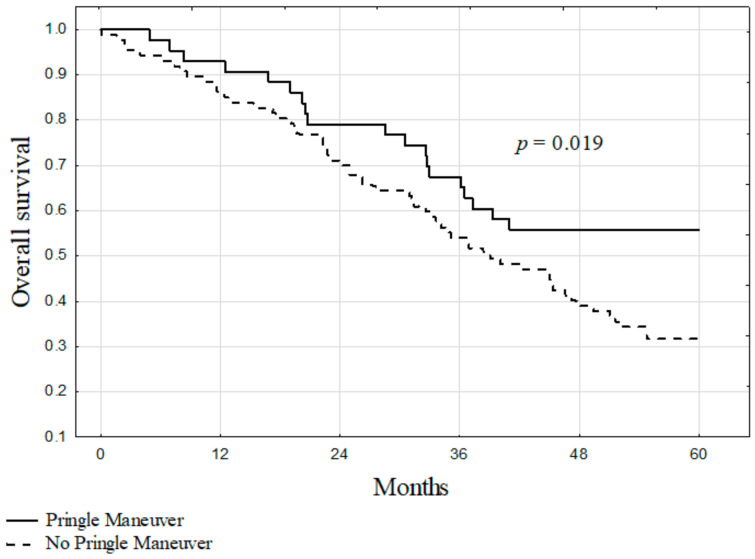
The overall survival of patients after liver resections for colorectal metastases with and without the Pringle maneuver in the entire cohort.

**Figure 2:**
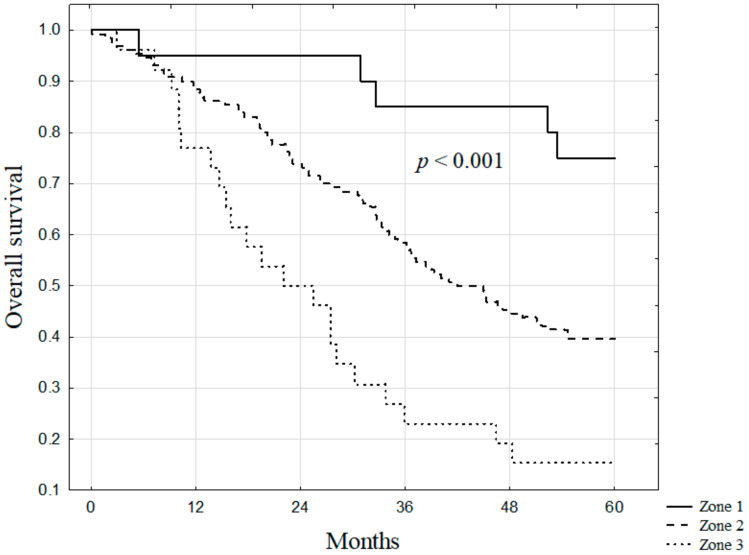
The overall survival of patients in the studied group stratified by Tumor Burden Score groups.

**Figure 3:**
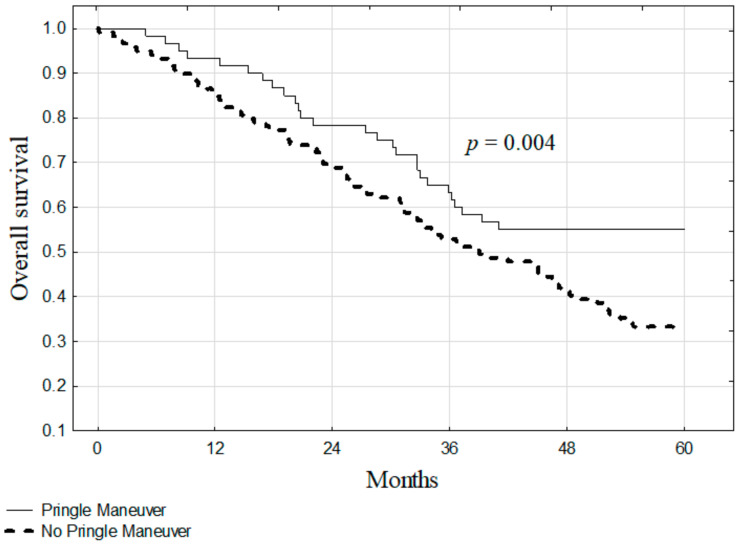
The overall survival of patients with or without the Pringle maneuver in Zone 2 of the Tumor Burden Score.

The median survival for all patients included in the study was 43 months. The 5-year overall survival was significantly higher in patients after liver resections with the PM (55.0%) as compared to those without the PM (33.4%, *p* = 0.019; Figure 1). The survival also differed significantly between patients in Zones 1, 2, and 3 (75.0%, 39.7%, and 15.4%, respectively) of TBS (*p* < 0.001; Figure 2). In subgroup analyses according to TBS, significantly higher survival after liver resections with the use of the PM was found for patients in Zone 2 (55.8% vs. 31.9%, *p* = 0.004; Figure 3). In Zones 1 and 3, survival after liver resections with the use of the PM (100.0% and 27.3%, respectively) was non-significantly higher than after operations without the PM (64.3% and 6.7%, respectively; *p* = 0.101 and *p* = 0.095, respectively).

**Figure 4:**
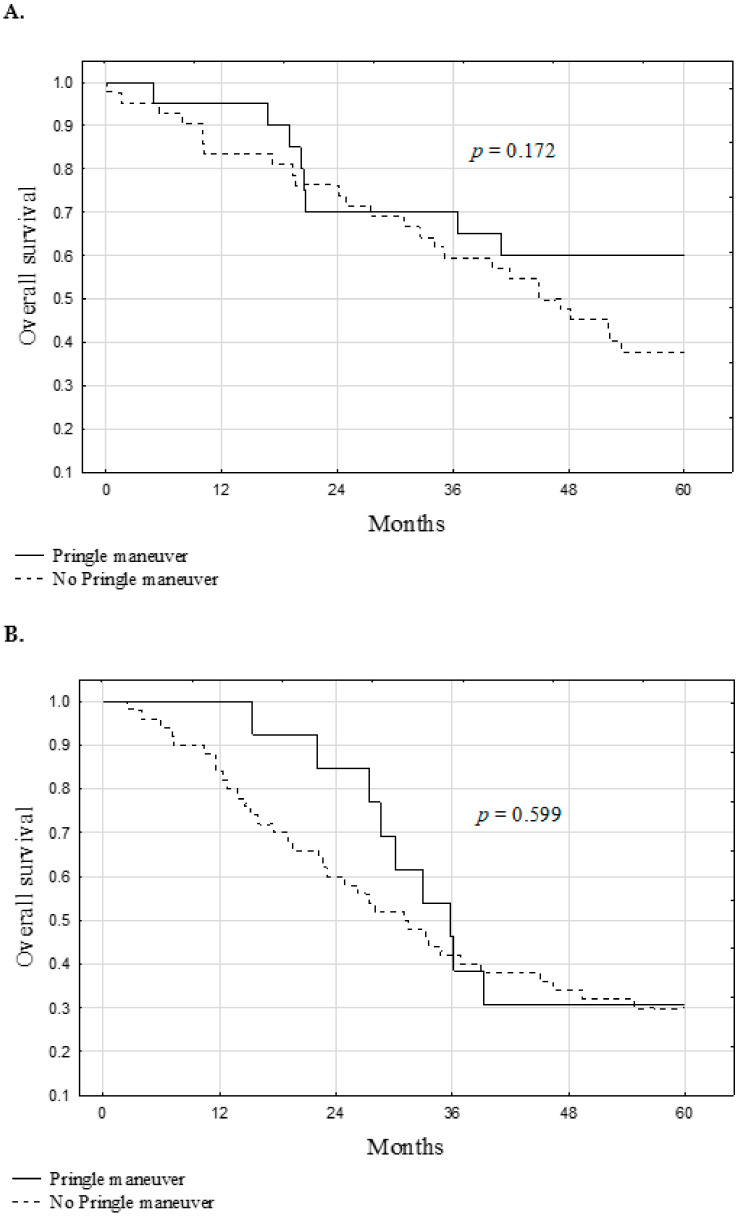
Overall survival after liver resection for colorectal metastases with and without Pringle maneuver in patients with preoperative carcinoembryonic antigen concentration (**A**) below median and (**B**) equal or higher than median.

Univariable analyses of the risk factors for worse overall survival are presented in Table 2. Significant risk factors comprised a higher number of tumors (*p* = 0.044), higher sum of all tumor diameters (*p* < 0.001), lower preoperative hemoglobin concentration (*p* = 0.009), higher preoperative CEA concentration (*p* = 0.029), and liver resections without the PM (*p* = 0.017). However, a series of two-factor analyses revealed that the association between the use of the PM and overall survival was not significant following adjustment for the effect of the preoperative CEA concentration (*p* = 0.265; Table 3). Further, in subgroup analyses performed after the division of patients according to the median preoperative CEA concentration, there were no significant differences in the overall survival between patients undergoing liver resection with and without the use of the PM (Figure 4).

## 4. Discussion

The use of the PM during elective liver resections due to colorectal metastases still raises controversies. These include, among others, the risk of post-hepatectomy liver failure, septic complications in the postoperative period, and the plausible impact on survival [10]. In this analysis of patients with colorectal liver metastases focused on long-term survival, no negative effects related to the use of the PM were found. Although the observed absolute survival outcomes were better after resections with the PM, this appears to be associated with the confounding effect of the lower preoperative CEA concentration in this group. 

Multiple studies have analyzed the early and late effects of hepatic pedicle clamping during hepatectomy [7,11,12,13]. First, the impact of the PM was studied in animal models. The results showed an increased growth of micrometastases of colon cancer when pedicle clamping was applied [8]. The concept was based upon ischemia/reperfusion injury and a higher metastatic potential of cancer cells in that setting. However, the studies made in the murine model were not backed up entirely by the analysis in the clinical settings. For instance, there is an ongoing debate regarding the utility of the PM in hepatocellular cancer (HCC) patients. 

The use of the PM in HCC patients is often applied due to concomitant cirrhosis and the risk of increased intraoperative blood loss. In the latest post-hoc analysis of two randomized studies, the authors found better overall survival with no effect on the disease-free survival for patients when the PM was used in cirrhotic patients [14]. Counterintuitively, blood loss is usually not diminished by the PM [15]. In our study, blood transfusion requirement was not dependent upon the use of pedicle clamping. Secondly, as no effect on disease-free survival was observed, the explanation of that phenomenon cannot be based upon tumor recurrence itself. 

In concordance, similar studies have been performed in colorectal patients. In 2010, Ferrero et al. stated the question of survival in patients with colorectal metastases in the settings of the PM [16]. The study was randomized and comprised eighty colorectal cancer patients. The use of the PM did not influence the overall survival. In comparison to our study, the cohort included various hepatectomies, however, with no differences in terms of the major hepatectomies rate between both groups. The analysis unexpectedly revealed higher transfusion rates in the PM group. Nevertheless, in the latest report, the blood transfusions were not found to be independently associated with worse survival in patients with colorectal liver metastases when early postoperative deaths were excluded [17]. 

In 2013, De Carlis et al. conducted a study including 120 patients with case-matched analysis of colorectal cancer metastases revealing significantly better recurrence-free survival with pedicle clamping [18]. The authors denoted significantly better recurrence-free survival (49.9% vs. 18.3%, *p* = 0.010) and with better, although non-significant, overall survival (47.2% vs. 32.1%, *p* = 0.06). In comparison to other studies, as the authors emphasized, to eliminate the confounding factors, the study group was highly selected and included only patients without risk factors for worse survival as identified by Fong Y et al. in his landmark study [19]. 

In our study, we proposed the TBS model to stratify the patients accordingly to low, moderate, and poor prognoses. The TBS had been recently introduced as a new concept to stratify the risk for colorectal metastatic patients [9]. In our study, we confirmed that a novel TBS provides a valuable prognostic model in patients with colorectal liver metastases. The model is able to stratify patients into three distinct groups as was shown on the Kaplan–Meier survival analysis. 

This scoring system is based solely upon the tumor characteristics. Interestingly, in our material, the subgroup analyses of patients stratified by TBS demonstrated significantly better survival within Zone 2 of TBS, and better, although non-significant, survival in Zone 1 and 2 when the PM was used. Nevertheless, these analyses appear highly biased by differences in the preoperative CEA concentration, as the significant protective effect of the PM disappeared following adjustment for the confounding role of CEA. 

In the study by De Carlis et al. the inclusion of PM patients was also only possible when 15 min clamping with 5 min of relapse was utilized not exceeding three times [18]. In our material, the PM time was highly heterogenous, and often applied for more than 15 min (median duration of 30 min). This is reflected by significantly higher peak postoperative bilirubin concentration following resections with the PM, although the observed difference is of limited clinical importance. In the latest report by the Al-Saeedi et al. from the University of Heidelberg group, the PM with a median time of 19 min was found to be associated with lower intraoperative bleeding and major morbidity risk [20]. 

There was no difference in the 3-year recurrence free-survival observed by the authors in the whole studied group comprising both primary and secondary liver tumors, as well as in the subgroup analysis of colorectal metastases. Even though the cohort comprised a heterogeneous group of patients, similarly to our study, the inclusion criteria were a major hepatectomy (four right liver segments or more resected). Likewise, in the matched cohort study by Tsang et al., no difference in the overall and recurrence-free survival was observed when the intermittent PM was applied for no longer than 15 min in colorectal patients [21]. 

One of the threats of the PM is the occurrence of IRI. Usually, the peak of ALT activity and bilirubin concentration is correlated with the PM duration [22]. Notably, in our analysis, neither of these factors were identified as risk factors for worse survival. Although the use of the PM did influence the postoperative peak bilirubin concentration, it did not influence the peak postoperative ALT activity.

A limitation of the current study is its retrospective nature, as no randomization was applied, and the PM was used solely depending on the surgeon’s decision. There was also no predetermined technique of the PM used, and both intermittent and continuous ones were used. Due to partially missing data from the follow-up, the recurrence-free survival could not be analyzed. In addition, the tumor mutation status was not routinely established in the period of our analysis, and we, unfortunately, do not have robust data on the response to chemotherapy for this retrospective cohort. The carcinoembryonic antigen was not evenly distributed among the PM and no-PM groups and was found to be an independent risk factor in bivariate analysis with the PM. This possible selection bias is restraining the authors from drawing strong conclusions identifying the superiority of the OS in the PM group.

## 5. Conclusions

In conclusion, the use of the PM had no negative effects on the long-term outcomes in patients undergoing major, oncologically radical liver resections for colorectal metastases.

## Figures and Tables

**Table 1:** Baseline characteristics of the study group and comparison of patients with and without the use Pringle maneuver during liver resection for colorectal liver metastases.

Variables	Number (%) or Median (IQR)	Pringle Group	No-Pringle Group	*p*
**Sex**				0.338
male	108 (60.3%)	38 (63.3%)	70 (58.8%)	
female	71 (39.7%)	22 (36.7%)	49 (41.2%)	
**Age** (years)	62 (56–70)	64 (57–72)	61 (55–69)	0.129
**Liver resection**				
right hemihepatectomy	128 (71.5%)	39 (65.0%)	89 (74.8%)	
extended right hemihepatectomy	20 (11.2%)	8 (13.3%)	12 (10.1%)	
right hemihepatectomy and non-anatomical resection	31 (17.3%)	13 (21.7%)	18 (15.1%)	
**Pringle maneuver**	60 (33.5%)			
**Pringle maneuver** (median duration [min])	30			
**Intraoperative blood transfusion** (PRBCs units)	0 (0–5) *	0 (0–5) *	0 (0–3)	0.474
**Number of tumors**	2 (1–3)	2 (1–4)	2 (1–3)	0.496
**Tumor Burden Score zone**				0.610
Zone 1		6 (10.0%)	14 (12.1%)	
Zone 2		43 (71.7%)	87 (75.0%)	
Zone 3		11 (18.3%)	15 (12.9%)	
**Diameter of the largest tumor** (mm)	45 (29–65)	48.5 (30.0–67.0)	41.0 (27.5–65.0)	0.533
**Tumor Burden Score** (quantitative)	5.40 (4.03–7.39)	6.08 (4.35–7.63)	5.01 (3.88–7.28)	0.132
**Preoperative laboratory results**				
White Blood Cell (10^3^/µL)	6.30 (4.80–7.78)	6.70 (5.30–9.04)	6.03 (4.70–7.32)	0.023
Hemoglobin (g/dL)	13.10 (11.70–14.00)	13.40 (11.50–14.00)	13.00 (11.90–14.00)	0.658
Platelets (10^3^/µL)	236 (184–292)	268 (220–311)	211 (176–262)	0.001
Aspartate aminotransferase (IU/L)	30 (22–41)	31 (20–47)	30 (23–39)	0.659
Alanine aminotransferase (IU/L)	25 (19–37)	23 (20–51)	25 (19–37)	0.592
Bilirubin (mg/dL)	0.56 (0.37–0.78)	0.47 (0.34–0.59)	0.61 (0.41–0.90)	0.001
Albumin (g/dL)	4.00 (3.70–4.20)	4.00 (3.60–4.40)	4.00 (3.70–4.15)	0.464
INR	0.99 (0.96–1.04)	1.01 (0.96–1.04)	0.99 (0.95–1.04)	0.836
Creatinine (mg/dL)	0.81 (0.71–0.94)	0.81 (0.76–0.93)	0.81 (0.68–0.94)	0.345
Carcinoembryonic antigen (ng/mL)	10.80 (3.30–49.50)	6.00 (2.00–19.00)	12.66 (4.00–62.15)	0.026
Carbohydrate antigen 19-9 (ng/mL)	20.00 (6.60–79.13)	27.00 (5.80–136.10)	15.60 (7.00–66.40)	0.357
Peak 3-day postoperative bilirubin (mg/dL)	2.29 (1.61–3.20)	2.71 (1.94–3.78)	2.06 (1.43–2.91)	0.002
Peak 3-day postoperative ALT (U/L)	334 (205–492)	315 (190–492)	343 (210–496)	0.415
Peak postoperative lactate (mmol/L)	3.85 (2.60–5.60)	4.70 (3.80–6.30)	3.60 (2.60–5.30)	0.113

* median (minimum–maximum); INR—International Normalized Ratio.

**Table 2:** Univariable analyses of the risk factors for worse overall survival after liver resection for colorectal metastases.

Pringle vs. No Pringle Maneuver		Covariate		
HR (95% CI)	*p*		HR (95% CI)	*p*
0.55 (0.36–0.86)	0.009	Number of tumors (per 1 tumor)	1.08 (1.01–1.15)	0.002
0.57 (0.37–0.88)	0.001	Sum of all tumor diameters (per 1 mm)	1.01 (1.00–1.02)	<0.001
0.57 (0.36–0.90)	0.002	Hemoglobin (per 1 g/dL)	0.83 (0.73–0.95)	0.001
0.73 (0.43–1.26)	0.265	CEA (per 1 ln[ng/mL])	1.31 (1.00–1.28)	0.049

**Table 3:** The two factor analysis of risk factors for worse overall survival after liver resection for colorectal metastases, including the Pringle maneuver and potential confounders.

	HR (95% CI)	*p* Value
Patient sex (male vs. female)	1.04 (0.70–1.53)	0.857
Age (per 1 year)	1.01 (0.99–1.03)	0.397
Number of tumors (per 1 tumor)	1.07 (1.00–1.15)	0.044
Sum of all tumor diameters (per 1 mm)	1.01 (1.01–1.02)	<0.001
Liver steatosis (yes vs. no)	0.77 (0.51–1.16)	0.209
White Blood Cell (per 10^3^/µL)	1.01 (0.93–1.89)	0.892
Bilirubin (per 1 mg/dL)	0.85 (0.59–1.23)	0.384
Hemoglobin (per 1 g/dL)	0.84 (0.74–0.96)	0.009
CEA (per 1 ln[ng/mL])	1.14 (1.01–1.29)	0.029
Peak 3-day postoperative ALT (per 1 ln[U/L])	1.16 (0.86–1.56)	0.327
Peak 3-day postoperative bilirubin (per 1 mg/dL)	1.02 (0.72–1.46)	0.897
Peak postoperative lactate (per 1 mmol/L)	1.03 (0.93–1.15)	0.542
No Pringle maneuver	1.71 (1.10–2.64)	0.017

## Data Availability

The datasets generated or analyzed during the current study are available from the corresponding author on reasonable request.
